# Locomotor performance parameters as predictors of high-performing male soccer teams. A multiple-season study on professional soccer

**DOI:** 10.1038/s41598-024-80181-z

**Published:** 2024-11-18

**Authors:** Piotr Makar, Rabiu Muazu Musa, Rui Miguel Silva, Jarosław Muracki, Robert Trybulski, Emre Altundağ, Murat Altaca, Wacław Kuczmik, Rafał Studnicki, Zeki Akyildiz

**Affiliations:** 1 Faculty of Physical Education, Gdańsk University of Physical Education and Sport, Gdańsk, Poland; 2 Centre for Fundamental and Continuing Education, Universiti Malaysia Terengganu, Kuala Nerus, Malaysia; 3 Escola Superior de Desporto e Lazer, Instituto Politécnico de Viana do Castelo, Viana do Castelo, Portugal; 4 Research Center in Sports Performance, Recreation, Innovation and Technology SPRINT, Melgaço, Portugal; 5 Institute of Physical Culture Sciences, Department of Physical Culture and Health, University of Szczecin, Szczecin, Poland; 6 Provita Żory Medical Center, Żory, Poland; 7 Medical Department, The Wojciech Korfanty Upper Silesian Academy, Katowice, Poland; 8 Sport Science Faculty, Kütahya Dumlupınar University, Kutahya, Turkey; 9 Football Industries MBA, University of Liverpool, Liverpool, UK; 10 Department and Clinic of General Surgery, Vascular Surgery, Angiology and Phlebology, Faculty of Medical Sciences in Katowice, Medical University of Silesia in Katowice, Katowice, Poland; 11 Department of Physiotherapy, Medical University of Gdańsk, Gdańsk, Poland; 12 Department of Coaching Education, Sports Sciences Faculty, Afyon Kocatepe University, Afyonkarahisar, Turkey

**Keywords:** Football, Match analysis, Sports performance, Physical demands, Physiology, Medical research, Musculoskeletal system

## Abstract

This study aims to explore the interplay between locomotor demands and goal differentials to better understand their combined influence on overall success. Spanning three competitive seasons within the male Turkish Super League, this study analyzed all participating teams across 124 matches. Locomotor demands, including total distance (m) covered (TD), distances covered (m) at different speed thresholds (0.21–2.0 m/s; 2.01–4.0 m/s; 4.01–5.5 m/s; and 5.5–7.7 m/s), and the number of accelerations in range of 5.5–7.0 m/s (n), were quantified using an optical tracking system. Subsequently, regression models were employed to predict the total points earned by all teams over the three seasons. The logistic regression model, tailored to predict team categorization as high-points earners (HPE) or low-points earners (LPE) based on locomotor variables, exhibited a mean accuracy of 74%. Notably, total distance covered, running speed intervals between 4.4 and 5.5 m/s, and the number of accelerations in range of 5.5–7.0 m/s emerged as significant predictors of team success. Our findings highlight the pivotal role of running speed (4.01–5.5 m/s), number of accelerations, and total distance in predicting success for high-performing teams. Coaches can leverage these insights to refine training programs, thereby optimizing team performance, and fostering success in competitive environments.

## Introduction

Soccer entails an intermittent and demanding physical regimen, substantiated by scientific investigations into the sport’s physiological requirements, emphasizing the substantial contribution of aerobic metabolism and anaerobic processes to sustain intensified efforts^1,2^. Players normally cover distances ranging between 10 and 14 km, with a notable portion executed at higher-intensity running speed^3^. The soccer game’s dynamic nature necessitates frequent bursts of sprinting, accelerations, and decelerations, placing significant strain on the musculoskeletal system^4^. The dynamic and chaotic characteristics of soccer, influenced by situational variables result in sudden changes in direction, demanding exceptional levels of agility, endurance, and anaerobic capacity^5^.

The literature underscores the relevant role of tactical and technical behavior in soccer emphasizing their profound impact on the physical demands of the sport^6^. Tactical behavior and technical proficiency are integral components that dictate the flow and outcome of a match and shape the locomotor, mechanical, and physiological demands imposed on players^7^. The interplay between tactical decisions and technical execution influences the spatial distribution of players on the field, strategic movements, and the intensity of physical exertion^8^. It is crucial to recognize that the inherent nature of the sport does not solely dictate the physical demands of soccer but is modulated by the tactical strategies employed by teams^8^.

Furthermore, these tactical behaviors are interwoven with contextual factors such as team dynamics, opponent strategies, and match-specific situations^9^. As a result, tactical decisions become a critical determinant of the physical demands placed on players^7^. The physical demands placed on players during a match significantly impact their ability to execute both collective and individual behaviors, sustain optimal performance levels, and overcome challenges posed by the opposing team^10,11^. Scrutinizing these physical metrics provides valuable insights into a team’s endurance, overall fitness, and capacity to maintain competitive intensity. In previous research^12^, the connection between team running performance and goal-scoring likelihood has been a focal point. Logistic regression analyses revealed a consistent trend, emphasizing the relevance of overall running distance in predicting a team’s success in scoring the first goal^12^. This relationship was notably more pronounced for total running distance compared to other metrics such as sprints or in-possession running^13^.

Artificial neural network models have also been employed to identify key physical attributes influencing a team’s success^14^. These attributes include the team’s average distance covered at specific speed intervals and the total distance covered without ball possession^14^. In a broader context, research into football leagues such as Serie A highlighted certain activities strongly linked to securing top positions in final rankings^15^. Increased sprint activity, goal attempts, total shots, shots on target, and assists were identified as critical factors contributing to a team’s heightened likelihood of achieving success^15^. Recent studies showed that positional roles exhibit distinct physical demands^16,17^. Defensive and central midfielders covered more total distance than other roles, while wide midfielders and forwards covered greater distances at higher intensities (≥ 20 and ≥ 25 km/h)^16,17^. Moreover, the second half of matches showed a reduction in total distance covered, particularly for attacking midfielders and wide players^16,17^.

Despite the above-mentioned research, none has delved into investigating how the locomotor demands of teams interact with goals scored and conceded to potentially elucidate the fluctuations in accumulated points by the teams. Such an inquiry could offer a more profound understanding of the significance of physical demands in determining the final performance in the league. If proven to be the case, this understanding could guide the translation of such insights into effective training plans and priorities. To our knowledge, no study has tested the combined impact of locomotor demands, goals scored and conceded on accumulated points by teams across multiple years within the same competition.

Therefore, this study aims to explore the final performance of teams (accumulated points throughout the season) and their variations concerning locomotor demands and goals scored and conceded. The investigation spans three years and focuses on the First Turkish male soccer league.

## Materials and methods

### Study design and participants

This study followed a longitudinal observational design. Match locomotor performance data were collected from three competitive seasons (2020/2023) within the male soccer Turkish Super League, encompassing all participating teams. A total of 124 matches were considered for analysis. Informed consent of the participants is not needed in this case. This study was approved by the Ethical Committee of Kütahya Dumlupınar University with decision number 02.2024–25. This study is aligned with the Declaration of Helsinki.

### Data collection

All matches of these teams were recorded with the Instat Sports optical tracking system, consisting of two cameras with 4 K resolution, a notebook, and the Instat software. The analysis of all locomotor demands during the matches was done automatically by Instat software. The Instat optical tracking system has been used for assessing locomotor demands in previous studies^18,19^. Upon connecting the cameras to the computer, the Instat software executed sharpness adjustments and calibration on the field image captured by the cameras, overseeing the acquired data.

The data included a total of 60 teams (20 per season) over three consecutive seasons. Each team played 38 matches per season, totalling 380 matches per season. For the statistical analysis, the locomotive performances achieved by each team in every match were gathered, amounting to a total of 1140 observations (380 × 3). Subsequently, the average performances of each team on a season-by-season basis and the total points earned by each team in every season were extracted, resulting in a final dataset of 60 data points (20 teams per season, i.e., one observation for each of the 20 teams). This procedure allowed us to gather additional information such as cumulative match outcomes (wins, losses, or draws), each team’s final rank per season, total points accumulated per season, and goals for/against. Since the total points earned per season determine which team wins the trophy, qualifies for the European football championship, and gets promoted or relegated, we are particularly interested in investigating the locomotive variables that contribute to the accumulation of these points in the current study.

Post device and software installation, a technician immediately conducted control procedures to ensure accurate data retrieval. To minimize error margins at critical points such as corners and crown points, technicians continually monitored the data flow. The cameras were strategically positioned within the live broadcast room, precisely at the midfield line level, providing a comprehensive field view in two sections. Following camera-computer integration, the Instat software streamlined the adjustment of image sharpness and camera calibration on the field. During the calibration process, the software guided the user to define a specific number of points as stipulated by the system. Upon encoding the team staff into the Scope software by an operator, the system autonomously initiated player tracking, recording the positional data of each player.

Given the proximity of players during corner kicks and set-piece situations, individual player location data was not automatically assigned by the system. Consequently, the operator assumed the responsibility of identifying players during such instances to ensure accurate player attribution and prevent data loss. The Scope software periodically prompted the operator with location-based queries to uphold the precision of optical tracking throughout the entirety of the match. Finally, the locomotor measures analyzed were comprised of the total distance (m) (TD), and distances covered at different speed thresholds (m) (0.21–2.0 m/s; 2,01–4.0 m/s; 4.01–5.5 m/s; and 5.51–7.0 m/s), and the number of accelerations in ranges from 5.5 to 7.0 m/s and exceeding 7.01 m/s, and maximal speed (m/s) were considered for further analysis. Stoppage moments were not excluded when collecting variables throughout the ninety minutes of the match. Moreover, the COVID season (2020–2021 season) included in the analysis was not affected as the league continued without interruptions. No match was excluded from the analysis.

### Statistical analysis

The normality of the variables was assessed using the Shapiro–Wilk test, indicating that all variables conformed to a normal distribution.

K-means clustering was utilized to categorize teams based on the cumulative points earned per season. An optimal number of clusters (k = 2) was determined using the Elbow method, which identifies the point where the within-cluster sum of squares (WSS) begins to diminish at a decreasing rate, offering valuable insights into the dynamics and characteristics of teams with analogous patterns within the league. A binary logistic regression model was applied to classify the two groups of teams based on the locomotor variables obtained. To assess the model’s performance, a stratified five-fold cross-validation technique was employed to ensure that each cluster was present during the training and testing of the model^20^. The data was split into a ratio of 70:30 for training and testing^21,22^. A total of 41 team observations were used to train the model, while the remaining 19 team observations were used for testing to evaluate the predictability of the model in determining the created clusters. During this stage, the selection of clusters in each phase of training and testing was automatically completed by the algorithm, ensuring a bias-free selection criterion.

The model’s performance was assessed through metrics such as accuracy (ACC), area under the curve (AUC), recall, precision (PREC), F1 score, Kappa, and Matthews Correlation Coefficient (MCC). ACC represents the correctly classified instances, AUC measures the model’s ability to distinguish between classes and recall and PREC highlights true positive ratios. The F1 score gauges the harmonic mean of PREC and recall, offering an average accuracy for both classes. Kappa adjusts for random accuracy, while MCC, a discrete version of Pearson’s correlation coefficient, quantifies binary classification quality. The confusion matrix was used to illustrate instances correctly and incorrectly classified between classes. Additionally, a significance attribute evaluation identified locomotor variables influencing model accuracy. Multivariable binary logistic regression pinpointed significant locomotor variables predicting team group levels. These metrics collectively evaluated the model’s efficacy in addressing classification challenges in this study. The Pycaret libraries were evoked for the development of the LR model via Spyder IDE. Other statistical analyses were employed using Statistica software (version 10.0; Statsoft, Inc., Tulsa, OK, USA). Significance for all analyses was set at *p* ≤ 0.05.

## Results

**Fig. 1:**
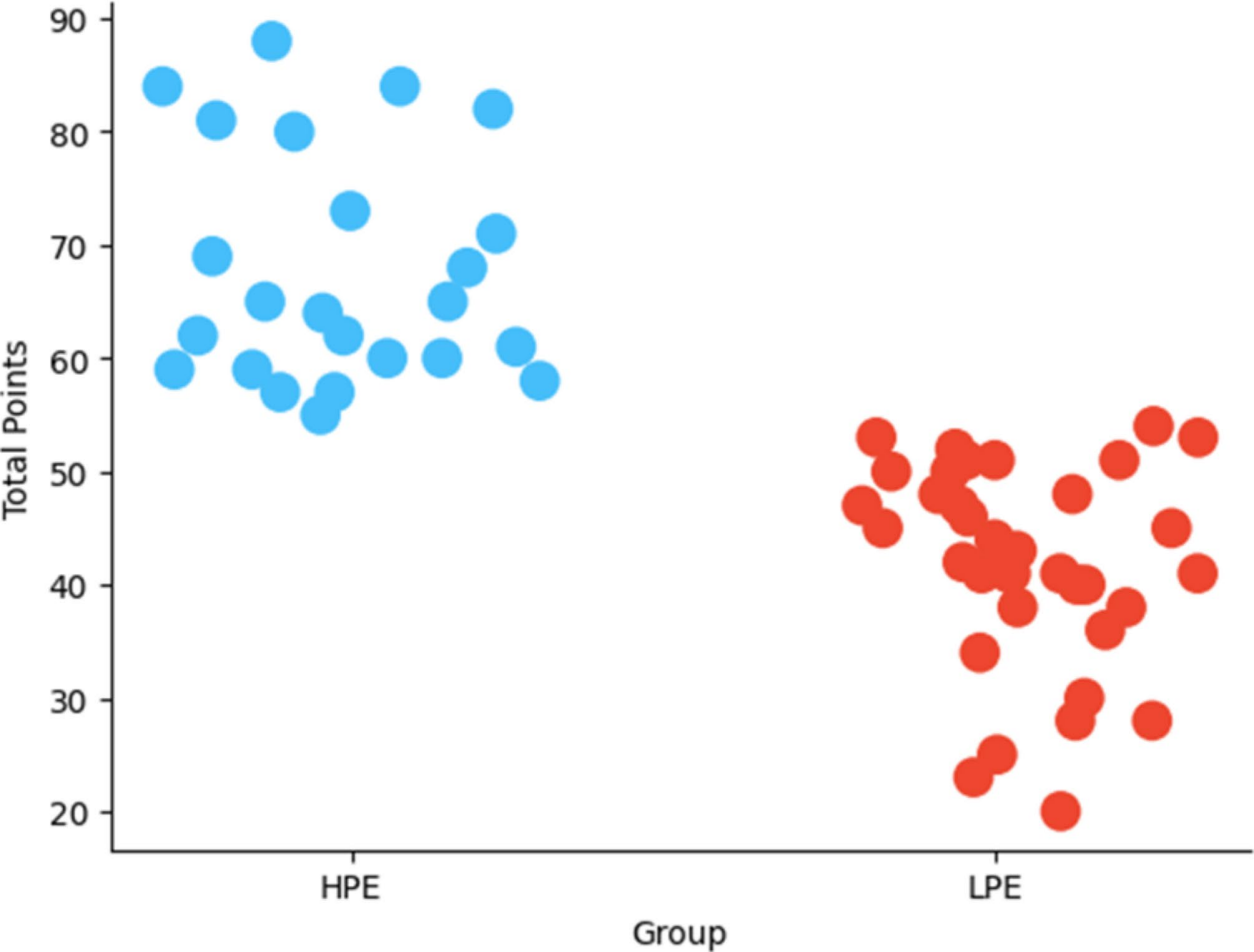
Grouping of the teams based on points earned.

Figure 1 illustrates the outcomes of the k-means clustering analysis, categorizing teams into two groups: high point earners (HPE) and low point earners (LPE). It is worth noting that the decision to group the teams into two clusters was based on the elbow method and silhouette score distance, which indicated that the two groups provided the most effective separation of the teams based on their points earned. A sum of 24 teams were classified as HPE and 36 teams were classified as LPE. As depicted, the HPE group boasts an average point score of 68, contrasting with the LPE group’s average score of 42.

Table 1 presents descriptive statistics for the investigated parameters, including locomotor variables, mean values, and standard deviations of the average locomotive performance from each group per season according to team rank. From the table, it can be seen that HPE teams have consistently finished the season in the top 4 positions, while LPE teams mostly occupy the bottom 3 and mid-table positions.

**Table 1:** Descriptive statistics of the study variables.

Locomotive variables	Season	HPE				LPE			
		Team rank	*N*	Mean	SD	Team rank	*N*	Mean	SD
Total distance (m)	1st	Bottom-3	0	N/A	N/A	Bottom-3	3	109357.70	2663.90
		Mid Table	5	109970.70	2055.34	Mid Table	8	110553.50	3009.44
		Top-4	4	110920.10	1250.63	Top-4	0	N/A	N/A
	2nd	Bottom-3	0	N/A	N/A	Bottom-3	4	110856.90	2950.77
		Mid Table	5	113245.80	2069.56	Mid Table	7	113242.30	1969.67
		Top-4	4	115228.20	2838.04	Top-4	0	N/A	N/A
	3rd	Bottom-3	0	N/A	N/A	Bottom-3	3	113197.30	1405.56
		Mid Table	2	117534.50	2186.37	Mid Table	11	113785.10	1817.21
		Top4	4	112986.40	584.25	Top4	0	N/A	N/A
Distance on speed up to 0.2 m/s (m)	1st	Bottom-3	0	N/A	N/A	Bottom-3	3	365.00	11.40
		Mid Table	5	357.95	12.67	Mid Table	8	346.610	21.61
		Top-4	4	359.73	14.27	Top-4	0	N/A	N/A
	2nd	Bottom-3	0	N/A	N/A	Bottom-3	4	354.62	19.98
		Mid Table	5	356.89	33.64	Mid Table	7	346.17	18.18
		Top-4	4	349.08	23.63	Top-4	0	N/A	N/A
	3rd	Bottom-3	0	N/A	N/A	Bottom-3	3	389.67	33.21
		Mid Table	2	381.58	28.77	Mid Table	11	381.47	25.51
		Top4	4	397.69	16.02	Top4	0	N/A	N/A
Distance on speed 0.21–2 m/s (m)	1st	Bottom-3	0	N/A	N/A	Bottom-3	3	42473.46	417.12
		Mid Table	5	40704.70	1283.21	Mid Table	8	41285.17	689.18
		Top-4	4	41161.49	488.56	Top-4	0	N/A	N/A
	2nd	Bottom-3	0	N/A	N/A	Bottom-3	4	41805.11	83.88
		Mid Table	5	40881.42	712.76	Mid Table	7	41145.22	653.07
		Top-4	4	41130.57	1018.58	Top-4	0	N/A	N/A
	3rd	Bottom-3	0	N/A	N/A	Bottom-3	3	41439.51	995.57
		Mid Table	2	41456.15	3095.82	Mid Table	11	41819.93	1299.44
		Top4	4	41487.25	1593.08	Top4	0	N/A	N/A
Distance on speed 2.01–4 m/s (m)	1st	Bottom-3	0	N/A	N/A	Bottom-3	3	40298.20	1602.00
		Mid Table	5	41396.53	1090.49	Mid Table	8	42246.45	2097.89
		Top-4	4	42354.78	686.21	Top-4	0	N/A	N/A
	2nd	Bottom-3	0	N/A	N/A	Bottom-3	4	41360.96	1652.15
		Mid Table	5	43429.71	1063.42	Mid Table	7	43083.53	1165.87
		Top-4	4	44220.16	2269.26	Top-4	0	N/A	N/A
	3rd	Bottom-3	0	N/A	N/A	Bottom-3	3	42843.54	1949.07
		Mid Table	2	45511.00	1531.27	Mid Table	11	43205.13	1259.53
		Top4	4	42690.10	826.10	Top4	0	N/A	N/A
Distance on speed 4.01–5,5 m/s (m)	1st	Bottom-3	0	N/A	N/A	Bottom-3	3	17342.98	730.57
		Mid Table	5	18100.78	670.57	Mid Table	8	17660.85	1213.72
		Top-4	4	17901.93	458.13	Top-4	0	N/A	N/A
	2nd	Bottom-3	0	N/A	N/A	Bottom-3	4	17750.14	1208.80
		Mid Table	5	18934.84	799.86	Mid Table	7	18748.78	850.19
		Top-4	4	19456.23	1256.34	Top-4	0	N/A	N/A
	3rd	Bottom-3	0	N/A	N/A	Bottom-3	3	18683.31	477.61
		Mid Table	2	20212.92	2378.82	Mid Table	11	18649.84	974.43
		Top4	4	18555.71	965.42	Top4	0	N/A	N/A
Distance on speed 5.51–7 m/s (m)	1st	Bottom-3	0	N/A	N/A	Bottom-3	3	7412.44	207.80
		Mid Table	5	7931.87	174.41	Mid Table	8	7514.01	565.75
		Top-4	4	7638.79	220.56	Top-4	0	N/A	N/A
	2nd	Bottom-3	0	N/A	N/A	Bottom-3	4	7975.16	726.57
		Mid Table	5	8088.76	384.94	Mid Table	7	8300.89	532.96
		Top-4	4	8433.65	416.28	Top-4	0	N/A	N/A
	3rd	Bottom-3	0	N/A	N/A	Bottom-3	3	8119.69	137.72
		Mid Table	2	8370.77	1187.29	Mid Table	11	8026.08	512.13
		Top4	4	8083.08	591.49	Top4	0	N/A	N/A
Distance on speed over 7 m/s (m)	1st	Bottom-3	0	N/A	N/A	Bottom-3	3	1488.35	4.577
		Mid Table	5	1500.20	139.81	Mid Table	8	1522.11	132.97
		Top-4	4	1511.22	85.48	Top-4	0	N/A	N/A
	2nd	Bottom-3	0	N/A	N/A	Bottom-3	4	1630.05	207.40
		Mid Table	5	1574.68	119.23	Mid Table	7	1639.63	166.27
		Top-4	4	1658.34	178.44	Top-4	0	N/A	N/A
	3rd	Bottom-3	0	N/A	N/A	Bottom-3	3	1744.03	191.50
		Mid Table	2	1621.96	217.08	Mid Table	11	1726.36	184.53
		Top4	4	1794.96	232.39	Top4	0	N/A	N/A
No. of accelerations (5.5–7 m/s) (n)	1st	Bottom-3	0	N/A	N/A	Bottom-3	3	490.50	15.37
		Mid Table	5	541.82	18.30	Mid Table	8	513.36	37.48
		Top-4	4	513.71	13.25	Top-4	0	N/A	N/A
	2nd	Bottom-3	0	N/A	N/A	Bottom-3	4	524.80	33.14
		Mid Table	5	536.11	21.64	Mid Table	7	546.90	31.35
		Top-4	4	548.45	34.67	Top-4	0	N/A	N/A
	3rd	Bottom-3	0	N/A	N/A	Bottom-3	3	538.49	7.29
		Mid Table	2	552.58	74.90	Mid Table	11	527.69	32.20
		Top4	4	520.40	26.86	Top4	0	N/A	N/A
No. of accelerations (> 7 m/s) (n)	1st	Bottom-3	0	N/A	N/A	Bottom-3	3	86.78	1.47
		Mid Table	5	87.53	6.76	Mid Table	8	87.23	8.02
		Top-4	4	87.25	3.52	Top-4	0	N/A	N/A
	2nd	Bottom-3	0	N/A	N/A	Bottom-3	4	94.08	10.35
		Mid Table	5	92.55	6.63	Mid Table	7	94.19	8.41
		Top-4	4	93.54	7.09	Top-4	0	N/A	N/A
	3rd	Bottom-3	0	N/A	N/A	Bottom-3	3	101.79	7.27
		Mid Table	2	97.73	15.07	Mid Table	11	102.45	10.66
		Top4	4	105.29	9.67	Top4	0	N/A	N/A
Maximal speed (m/s)	1st	Bottom-3	0	N/A	N/A	Bottom-3	3	8.09	0.05
		Mid Table	5	8.12	0.08	Mid Table	8	8.15	0.06
		Top-4	4	8.17	0.03	Top-4	0	N/A	N/A
	2nd	Bottom-3	0	N/A	N/A	Bottom-3	4	8.20	0.10
		Mid Table	5	8.17	0.04	Mid Table	7	8.19	0.08
		Top-4	4	8.21	0.09	Top-4	0	N/A	N/A
	3rd	Bottom-3	0	N/A	N/A	Bottom-3	3	8.28	0.08
		Mid Table	2	8.22	0.08	Mid Table	11	8.23	0.08
		Top4	4	8.25	0.08	Top4	0	N/A	N/A

*N/A* Not Available, *SD* Standard Deviation.

Table 2 summarizes the model’s performance in predicting team categorization as HPE or LPE based on locomotor variables. The model achieved a mean accuracy of 74%, indicating effective grouping prediction. The AUC was 0.71, demonstrating reasonable predictive modelling. PREC and Recall scores were 0.89 and 0.70, respectively, revealing accurate identification of positive cases. The Kappa score was 0.42, signifying reasonable reliability, and MCC showed a correlation of 0.46.

**Table 2:** Performance evaluation of the model for predicting HPE or LPE based on locomotor variables.

	Accuracy	AUC	Recall	PREC	F1	Kappa	MCC
Mean	0.7350	0.7083	0.7000	0.8917	0.7490	0.4152	0.4563
SD	0.1988	0.3146	0.2667	0.1750	0.2007	0.4482	0.4442

**Fig. 2:**
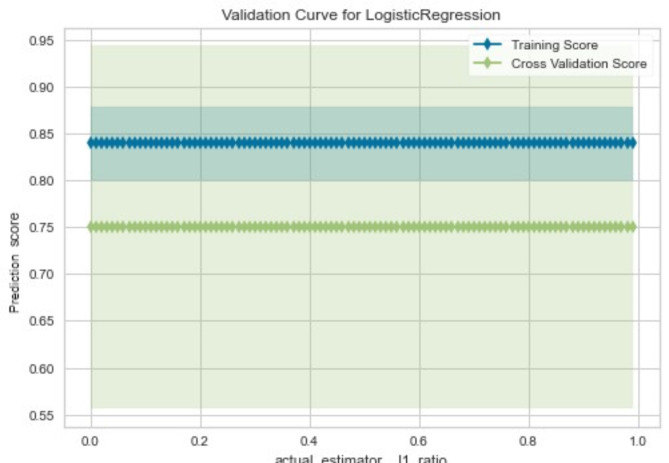
Performance of the model on test data.

Figure 2 illustrates the training and cross-validation scores of the model on the test dataset. The x-axis represents the ratio of L1 regularization (also known as Lasso) used in the logistic regression model. The L1 regularization helps in feature selection by penalizing the absolute value of the coefficients thereby effectively shrinking some coefficients to zero. The Y-axis reflects the accuracy of the model across different L1 regularization ratios. The training score, depicted at 0.83, exceeded the cross-validation score of 0.74. The model was, however, adjusted during the cross-validation stage, where the prediction accuracy score yielded 74%. The straight line observed in the gap between the training and cross-validation reflects that changing the L1 ratio does not significantly affect the model’s generalization ability against unseen data. This is essential in the visualization of the model performance as we checked the performances of the model across different regularization ratios. This suggests that the model performs very well on the training and cross-validation data regardless of the L1 ratio used.

**Fig. 3:**
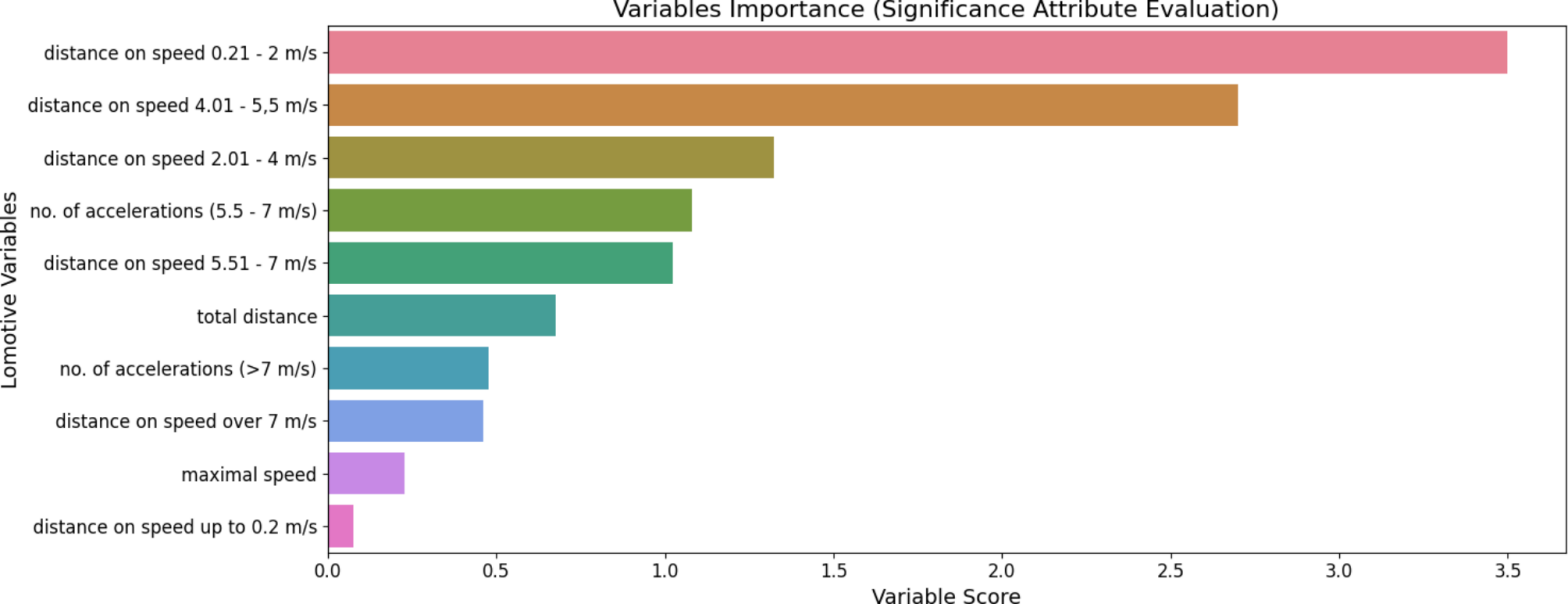
Locomotor variables contribution towards the model performance.

Figure 3 presents a graphical visualization of the variable contributions to the model pipeline performance through feature importance plots. Significance attribute evaluation identified locomotor variables that significantly influence model performance. Notably, 6 out of the 10 variables demonstrated contributions exceeding 50%, underscoring their importance in classifying teams as HPE or LPE. Further analysis involved multivariate logistic regression to ascertain the impact of these variables on team classification probability via odds ratio analysis. The use of multivariate logistic regression at this stage was necessary to study the likelihood of these 6 variables affecting points accumulation by the teams, a determination that could not be achieved through the machine-learning-based logistic regression model, which was primarily applied to predict or classify teams based on their points earned.

Table 3 summarizes the multivariable binary logistic regression model used to identify significant locomotor variables predicting team classification. The model exhibited good fit (Hosmer-Lemeshow > 0.05), a 65% accuracy, and notable discriminant capacity (AUC: 72% at 95% confidence). The model explains 22% of the variance in team categorization (Nagelkerke R^2^ = 0.22). The analysis revealed three significant predictors (*p* ≤ 0.05) in the multivariable model: distance covered at a speed of 4.01–5.5 m/s, number of accelerations (5.5–7 m/s), and total distance.

An increase of one unit in distance covered at a speed of 4.01–5.5 m/s during a game was associated with a 0.7% decrease in the likelihood of being classified as HPE (OR = 0.993, 95% CI = [0.986, 0.999]). Conversely, an increase of one unit in the number of accelerations (5.5–7 m/s) raised the likelihood of being classified as HPE by 8.3% (OR = 1.083, 95% CI = [0.999, 1.175]). Additionally, an increase of one unit in total distance covered was associated with a 0.6% increase in the likelihood of being classified as HPE (OR = 1.006, 95% CI = [1.000, 1.013]). Other variables did not show significant effects on the odds of being classified as HPE or LPE (*p* > 0.05).

**Table 3:** A multivariate analysis of significant locomotor variables determining high or low points earners.

Predictors	Estimate	SE	Z	p	OR	95% CI	
						Lower	Upper
Intercept	−37.550	23.739	−1.58	0.114	4.917	3.037	79.057
Dist. on speed 0.21–2 m/s (m)	−0.006	0.003	−1.78	0.075	0.994	0.988	1.001
Dist. on speed 2.01–4 m/s (m)	−0.006	0.003	−1.86	0.063	0.994	0.987	1.000
Dist. on speed 4.01–5.5 m/s (m)	−0.007	0.003	−2.19	0.029*	0.993	0.986	0.999
Dist. on speed 5.51–7 m/s (m)	−0.010	0.006	−1.79	0.073	0.99	0.979	1.001
no. of acc. in range (5.5–7 m/s) (n)	0.080	0.041	1.94	0.050*	1.083	0.999	1.175
total distance (m)	0.006	0.003	1.94	0.050*	1.006	1.000	1.013

*p ≤ 0.05; Nagelkerke R^2^ = 0.22; Hosmer Lemeshow (*p* = 0.96); AC = 65%; AUC = 0.72, OR = Odds Ratio, CI = Class Interval.

## Discussion

The primary objective of this study was to explore the interplay between locomotor demands, goals scored, and goals conceded in professional soccer, and its impact on the cumulative points garnered throughout a soccer season. The main findings revealed that locomotor patterns distinguish high-point earners (HPE) and low-point earners (LPE). Teams in the HPE category exhibited superior performance in specific locomotor measures, including total distance covered, running at different speed intervals, and acceleration.

Considering the locomotor measures and team performance, our model exhibited a commendable mean accuracy of 74%. Six locomotor variables emerged as significant contributors to the model’s predictive accuracy, encompassing total distance covered, speed at various intervals, and the number of accelerations. Specifically, teams displaying higher numbers of accelerations (5.5–7 m/s) during a game were more likely to go towards the HPE category. However, an increase in the distance covered in the speed zone of 4.01–5.5 m/s was associated with a decrease in the likelihood of being classified as HPE. The findings of the present study align with a previous study that also conducted a logistic regression, and demonstrated that the best model exhibited a mean accuracy of 77%, indicating a robust relationship between running performance and the likelihood of being a high-point earner^12^.

Specifically, this relationship was most pronounced for total running distance compared to high-speed, sprint, or in-possession running distance^12^. Moreover, in a previous study, a significant positive correlation was observed between high-intensity locomotor measures and team success, where success was defined by match outcomes (wins or losses) and specific performance metrics such as the number of goals scored^14^. Notably, a correlation exists between intense actions and critical moments in a match. For instance, high-intensity efforts, such as fast movements or sprints, frequently precede the majority of open-play goals^23^. However, our study pinpointed specific speed intervals and acceleration thresholds intricately associated with heightened team performance.

It was previously identified that the greatest predictors of the HPE team’s success were the average distance covered by the team at speeds between 15 and 20 km/h (99% weighting as a predictor), the total team-covered distance without the ball (76.8% weighting), and the average distance covered by the team (75.1% weighting)^14^. In contrast, a previous investigation has affirmed that teams demonstrating success have been characterized by their ability to cover substantial distances at reduced speeds while concurrently maintaining ball possession through strategic deployment of measured passes^24^. Furthermore, the volume of distance covered in the variables analyzed did not relate to success in soccer, independently of the team’s ranking^25^. Also, less and high-ranked teams presented the same running requirements at higher speed^25^. On the other hand, in previous studies, less ranked teams were those covering extended overall distances and distances surpassing 14 and 19 km/h, as well as distances exceeding 19.8 and 25.2 km/h^26,27^.

The observed disparities in previous studies regarding the relationship between high-intensity running and team success may be attributed to variations in tactical approaches, playing styles, and team strategies across different leagues and competitions^28,29^. The contrasting evidence in the literature may stem from these contextual intricacies, as teams with different playing styles and strategies may prioritize certain locomotor variables over others. For instance, the home or away status of matches might affect team performance, as playing at home can provide a psychological advantage and different physical demands compared to away matches^30^. Additionally, the match outcome itself (win, loss, or draw) could influence locomotor performance, with teams possibly exhibiting different physical efforts during the match^31,32^. Existing literature also highlights other contextual factors such as the quality of the opposition, tactical formations, and individual player roles, all of which could impact the locomotor variables prioritized by different teams^33^. In the context of the present study, the identified locomotor variables associated with team success include running speed intervals ranging from 4.01 to 5.5 m/s and the number of accelerations.

It is essential to acknowledge the limitations of our study. One of the main limitations is related to the fact that there is no study testing the Instat sports optical tracking system for its validity and reliability. Given that, our results should be taken with caution and future studies should test the Instat sports optical tracking system validity and reliability. While our logistic regression model performed well, limitations include the potential influence of contextual factors not captured in our analysis. Additionally, the relatively small dataset demands cautious generalization of our findings. The lack of discrimination of variables by specific player positions, restricts the generalizability and applicability of our findings to different player roles within the team. Given that, future research could explore the role of player positions in influencing these variables, thereby offering a better understanding of the individual player roles and team performance. Finally, the fact that we did not account for periods when the ball is out of play during the 90-minute game duration, could potentially affect the results.

Despite these limitations, our study offers valuable insights for practitioners. Coaches can design training regimes to emphasize locomotor metrics identified as significant predictors of team success. The identification of specific speed intervals and acceleration thresholds provides actionable information for optimizing team performance. Future research should focus on standardized methodologies, such as consistent definitions of speed zones, uniform data collection protocols (e.g., using the optical tracking devices and data processing software), and standardized match conditions (e.g., accounting for home/away status, opponent quality, and match outcomes).

## Conclusions

The main findings of the present study showed that running speed from 4.01 to 5.5 m/s, accelerations, and total distance were key predictors of high-point earner teams. The identification of such key predictors of successful teams offers coaches actionable metrics to enhance team performance. Coaches can leverage this evidence for effective training programming, focusing on these locomotor metrics to increase the team’s success.

## Limitations of the study

As a main limitation of this study we consider the lack of the contextual analysis. In future research the contextual factors such as winning, drawing and loosing conditions, deffensive, offensive or transition phase of the game should be considered. In this manuscript there is the number of accelerations in speed range of 5.5–7.0 and over 7.01 m/s [n] analyzed, but the parameters of minimal time and minimal acceleration threshold of this actions were not defined. We are aware that this is a methodological limitation and we would like to highlight the need of standardisation of the research protocols for conducting measurements of the running parameters in sports.

## Data Availability

Data used in this article is publicly available at this link: https://osf.io/whdsa/.
