# Author Correction: Locomotor performance parameters as predictors of high-performing male soccer teams. A multiple-season study on professional soccer

**DOI:** 10.1038/s41598-025-86900-4

**Published:** 2025-01-28

**Authors:** Piotr Makar, Rabiu Muazu Musa, Rui Miguel Silva, Jarosław Muracki, Robert Trybulski, Emre Altundağ, Murat Altaca, Wacław Kuczmik, Rafał Studnicki, Zeki Akyildiz

**Affiliations:** 1 Faculty of Physical Education, Gdańsk University of Physical Education and Sport, Gdańsk, Poland; 2 Centre for Fundamental and Continuing Education, Universiti Malaysia Terengganu, Kuala Nerus, Malaysia; 3 Escola Superior de Desporto e Lazer, Instituto Politécnico de Viana do Castelo, Viana do Castelo, Portugal; 4 Research Center in Sports Performance, Recreation, Innovation and Technology SPRINT, Melgaço, Portugal; 5 Institute of Physical Culture Sciences, Department of Physical Culture and Health, University of Szczecin, Szczecin, Poland; 6 Provita Żory Medical Center, Żory, Poland; 7 Medical Department, The Wojciech Korfanty Upper Silesian Academy, Katowice, Poland; 8 Sport Science Faculty, Kütahya Dumlupınar University, Kutahya, Turkey; 9 Football Industries MBA, University of Liverpool, Liverpool, UK; 10 Department and Clinic of General Surgery, Vascular Surgery, Angiology and Phlebology, Faculty of Medical Sciences in Katowice, Medical University of Silesia in Katowice, Katowice, Poland; 11 Department of Physiotherapy, Medical University of Gdańsk, Gdańsk, Poland; 12 Department of Coaching Education, Sports Sciences Faculty, Afyon Kocatepe University, Afyonkarahisar, Turkey

Correction to: *Scientific Reports* 10.1038/s41598-024-80181-z, published online 18 November 2024

The original version of this Article contained an error in the spelling of the author Zeki Akyildiz, which was incorrectly given as Zeki Akildiz.

The original Article has been corrected.

